# Assessment of Adverse Reactions, Antibody Patterns, and 12-month Outcomes in the Mother-Infant Dyad After COVID-19 mRNA Vaccination in Pregnancy

**DOI:** 10.1001/jamanetworkopen.2023.23405

**Published:** 2023-07-14

**Authors:** Arianna G. Cassidy, Lin Li, Yarden Golan, Caryl Gay, Christine Y. Lin, Unurzul Jigmeddagva, Megan A. Chidboy, Mikias Ilala, Sirirak Buarpung, Veronica J. Gonzalez, Emilia Basilio, Meghan Duck, Amy P. Murtha, Alan H. B. Wu, Kara L. Lynch, Ifeyinwa V. Asiodu, Mary K. Prahl, Stephanie L. Gaw

**Affiliations:** 1 Division of Maternal-Fetal Medicine, Department of Obstetrics, Gynecology & Reproductive Sciences, University of California, San Francisco; 2 Department of Bioengineering and Therapeutic Sciences, University of California, San Francisco; 3 Institute for Human Genetics, University of California, San Francisco; 4 Department of Family Health Care Nursing, University of California, San Francisco; 5 Center for Reproductive Sciences, Department of Obstetrics, Gynecology & Reproductive Sciences, University of California, San Francisco; 6 Division of Experimental Medicine, Department of Medicine, University of California, San Francisco, San Francisco, California; 7 Division of Pediatric Infectious Diseases and Global Health, Department of Pediatrics, University of California, San Francisco; 8 UCSF Benioff Children’s Hospital, University of California, San Francisco; 9 Department of Laboratory Medicine, University of California, San Francisco

## Abstract

**Importance:**

Longitudinal data on COVID-19 messenger RNA (mRNA) vaccine reactogenicity and immunogenicity in pregnancy and for the mother-infant dyad are needed.

**Objective:**

To examine COVID-19 mRNA vaccine reactogenicity and immunogenicity in pregnancy and observe longitudinal maternal and infant outcomes.

**Design, Setting, and Participants:**

This prospective cohort study of pregnant individuals enrolled in the COVID-19 Vaccination in Pregnancy and Lactation study from December 1, 2020, through December 31, 2021, with follow-up through March 31, 2022, was conducted at a large academic medical center in an urban metropolitan area in California. Pregnant individuals receiving COVID-19 mRNA vaccines (mRNA-1273 [Moderna] and BNT162b2 [Pfizer-BioNTech]) were eligible. Of 81 participants enrolled, 5 were excluded after enrollment: 1 terminated pregnancy, 1 received the third vaccine dose prior to delivery, and 3 delivered prior to completing the initial vaccine series.

**Exposure:**

COVID-19 mRNA vaccination at any time during pregnancy.

**Main Outcomes and Measures:**

The primary outcomes were vaccine response as measured by blood Immunoglobulin G (IgG) titers after each vaccine dose and self-reported postvaccination symptoms. Patients’ IgG titers were measured in cord blood and in infant blood at intervals up to 1 year of life; IgG and IgA titers were measured in maternal milk. Clinical outcomes were collected from medical records.

**Results:**

Of 76 pregnant individuals included in final analyses (median [IQR] maternal age, 35 [29-41] years; 51 [67.1%] White; 28 [36.8%] primigravid; 37 [48.7%] nulliparous), 42 (55.3%) received BNT162b2 and 34 (44.7%) received mRNA-1237. There were no significant differences in maternal characteristics between the 2 vaccine groups. Systemic symptoms were more common after receipt of the second vaccine dose than after the first dose (42 of 59 [71.2%] vs 26 of 59 [44.1%]; *P* = .007) and after mRNA-1237 than after BNT162b2 (25 of 27 [92.6%] vs 17 of 32 53.1%; *P* = .001). Systemic symptoms were associated with 65.6% higher median IgG titers than no symptoms after the second vaccine dose (median [IQR], 2596 [1840-4455] vs 1568 [1114-4518] RFU; *P* = .007); mean cord titers in individuals with local or systemic symptoms were 6.3-fold higher than in individuals without symptoms. Vaccination in all trimesters elicited a robust maternal IgG response. The IgG transfer ratio was highest among individuals vaccinated in the second trimester. Anti-SARS-CoV-2 IgG was detectable in cord blood regardless of vaccination trimester. In milk, IgG and IgA titers remained above the positive cutoff for at least 5-6 months after birth, and infants of mothers vaccinated in the second and third trimesters had positive IgG titers for at least 5 to 6 months of life. There were no vaccine-attributable adverse perinatal outcomes.

**Conclusions and Relevance:**

The findings of this cohort study suggest that mRNA COVID-19 vaccination in pregnancy provokes a robust IgG response for the mother-infant dyad for approximately 6 months after birth. Postvaccination symptoms may indicate a more robust immune response, without adverse maternal, fetal, or neonatal outcomes.

## Key Points

**Question:**

What is the association between reactogenicity and immunogenicity of mRNA COVID-19 vaccines in pregnancy?

**Findings:**

In this cohort study of 76 individuals who received messenger RNA (mRNA) COVID-19 vaccines in pregnancy, systemic symptoms after vaccination were associated with 90% higher IgG titers after the second dose, and symptoms were associated with 6.3-fold higher cord blood IgG titers than in individuals without symptoms; there were no adverse outcomes. Offspring had positive IgG titers for at least 5 to 6 months of life.

**Meaning:**

These results suggest that mRNA COVID-19 vaccines in pregnancy provoke an IgG response for the mother-infant dyad for 6 months after birth, and that systemic symptoms after vaccination may indicate a more robust immune response, without adverse outcomes.

## Teaser

This cohort study examines reactogenicity after receipt of up to 3 doses of COVID-19 mRNA vaccine in pregnant individuals and immunogenicity for up to 12 months in mother-infant dyads.

## Introduction

Growing evidence has demonstrated the safety and efficacy of COVID-19 messenger RNA (mRNA) vaccination in pregnancy.^1,2,3,4,5,6^ Infection with SARS-CoV-2 in pregnancy is associated with increased risk of severe illness and death and pregnancy-associated complications.^3,7,8,9,10,11,12,13,14,15^ COVID-19 vaccination before, during, and after pregnancy is recommended by public health authorities and professional organizations. However, knowledge gaps remain.

Pregnant individuals may worry about the theoretical risks of vaccine adverse effects, especially fever, on the developing fetus.^16^ Conversely, lack of symptoms may lead a vaccine recipient to question whether the vaccine is working. Although passively transferred antibodies after maternal vaccination have been shown to protect the infant against COVID-19 for up to 6 months of life,^17,18,19^ there are few data on longitudinal, linked outcomes for mother-infant dyads from pregnancy through the first year of life.

We sought to examine associations between reactogenicity and immunogenicity of COVID-19 mRNA vaccination in each trimester of pregnancy as well as antibody durability and clinical outcomes of mother-infant dyads until 1 year after birth.

## Methods

This was a prospective cohort study of pregnant individuals in the COVID-19 Vaccine in Pregnancy and Lactation study.^6,20,21^ The institutional review board of the University of California, San Francisco, approved this study. Participants at a large academic medical center were recruited through self-referral and prenatal clinics. Eligible participants were pregnant, planning to receive any COVID-19 mRNA vaccine (mRNA-1273 from Moderna or BNT162b2 from Pfizer-BioNTech) at any point in pregnancy, and willing to donate blood samples. Written, informed consent was obtained from all participants. We assessed participants enrolled from December 1, 2020, to December 31, 2021, and maternal-infant outcomes through March 31, 2022. Participants received whichever COVID-19 vaccine was available at the site of vaccine administration. The Strengthening the Reporting of Observational Studies in Epidemiology (STROBE) reporting guideline for cohort studies was followed in the preparation of this manuscript.

Only individuals who had received COVID-19 mRNA vaccines were included for analysis. Participants were excluded if they had not received a second vaccine dose at least 2 weeks prior to delivery.

### Clinical Data Collection

The following information was obtained from the medical record: maternal age at vaccination, vaccine manufacturer, dates of vaccination, gestational age at first vaccine dose, gravidity, parity, participant-reported race and ethnicity, prepregnancy body mass index (BMI; calculated as weight in kilograms divided by height in meters squared), pregnancy complications (preeclampsia or gestational hypertension, gestational diabetes, chorioamnionitis), COVID-19 diagnosis, gestational age at delivery, mode of delivery, infant Apgar scores, birth weight, and infant hospitalization data. Race and ethnicity data were collected because there are known racial and ethnic disparities in COVID-19 disease and vaccination in pregnancy.^22,23^ Detailed descriptions of clinical data are given in the eMethods in Supplement 1.

An online questionnaire on vaccine adverse effects was sent to all participants 28 days after each vaccine dose (eMethods in Supplement 1). Additional surveys were administered periodically to gather data on booster COVID-19 vaccine doses and breakthrough COVID-19 infections. Questionnaires were distributed using REDCap (Research Electronic Data Capture), a secure, web-based software platform designed to support data capture for research.^24,25^

### Sample Collection

Maternal blood samples were collected at 4 time points: (1) before vaccination (within 1 day of first dose); (2) after first dose (just prior to the administration of the second dose); (3) after second dose (4-10 weeks after second dose); and (4) after third dose (4-10 weeks after third dose). Maternal blood and cord blood samples were collected at delivery. Infant blood samples were collected with parental consent at the following time points: 6 to 8 weeks, 3 to 4 months, 5 to 6 months, and 9 to 12 months of life. Colostrum samples were collected on the infant’s first day of life. Human milk samples were self-collected by participants into sterile containers at the same time points infant blood samples were collected, when possible.

### Measurement of SARS-CoV-2–Specific IgM and IgG in Plasma Samples

Whole blood was collected into tubes containing EDTA, and plasma was isolated by centrifugation and immediately cryopreserved at −80 °C until analysis. Plasma immunoglobulin M (IgM) and IgG antibodies against spike receptor binding domain and nucleocapsid protein were measured using the Pylon 3D fluorescence-based fully automated immunoassay system (ET Healthcare) (eMethods in Supplement 1).^26^ The background-corrected signal of SARS-CoV-2 specific IgM and IgG antibodies was reported as relative fluorescent units (RFU); measurements higher than 50 RFU were considered positive.

### Measurement of SARS-CoV-2–Specific IgG and IgA in Milk Samples

Milk samples were either processed immediately by study staff or frozen by mothers in their home freezer immediately after pumping and transferred on ice to the laboratory for processing. Milk was aliquoted and stored at −80 °C until analyzed. An antispike enzyme-linked immunosorbent assay (Euroimmune) was used to measure IgA or IgG levels in human milk samples using the manufacturer’s protocol with minor modifications (eMethods in Supplement 1)^20,21^ Optical density values of samples were calculated by dividing by the provided calibrator optical density value; values with a sample:calibrator ratio higher than 1 were considered positive. Milk samples were analyzed in duplicate.

### Statistical analysis

For antibody analyses, we compared IgG titers at each time point by trimester of initial vaccine dose. We compared maternal IgG and cord IgG titers at delivery, grouped by trimester of initial vaccine dose. To assess the efficiency of passive antibody transfer from the mother to the fetus, transplacental antibody transfer ratios were calculated by dividing the cord blood IgG titer by the maternal IgG titer at the time of delivery and compared by trimester of vaccination. Transplacental antibody transfer ratios are a measure of degree of efficiency of IgG passage from maternal circulation through the placenta to the fetus.

We compared median IgG titers after the second vaccination among individuals with or without postvaccination symptoms. We compared incidence of postvaccination symptoms by vaccine manufacturer. We compared infant IgG titers and colostrum or milk IgA and IgG titers across time grouped by trimester of initial maternal vaccination.

Data analyses were conducted using Stata statistical software, version 14 (StataCorp LLC). Descriptive statistics included frequencies for categorical variables, and means, SDs, medians, and ranges for continuous variables. Group differences in categorical variables were analyzed using the Fisher exact test, and group differences in continuous variables were analyzed using Mann-Whitney *U* tests. Differences in symptom frequencies after each dose were evaluated with McNemar tests. The magnitude of associations between continuous variables was assessed with Spearman correlations. Nonnormal distributions and small group sizes were accommodated with nonparametric tests.

Sensitivity analyses were performed for postvaccination symptoms. These analyses excluded participants with COVID-19 infection or with a third vaccine dose during pregnancy, and participants whose IgG titer after the second vaccination was determined in samples collected after delivery.

The analyses were exploratory and did not involve specific hypotheses or adjustment for multiple comparisons. A 2-sided *P* < .05 was considered statistically significant.

The 30-day rate of the IgG titer decline expressed as a percentage per month was calculated between 2 blood collections separated by 2 months, using the following formula:

[(IgG*_s_* − IgG*_f_*)/(Day*_s_* − Day*_f_*)] × 30,

where IgG*_s_* and IgG_f_ indicate second and first titer results, respectively, and Day*_s_* and Day*_f_* indicate second and first date of blood draw, respectively.^26^

## Results

### Study Population

**Figure 1.**
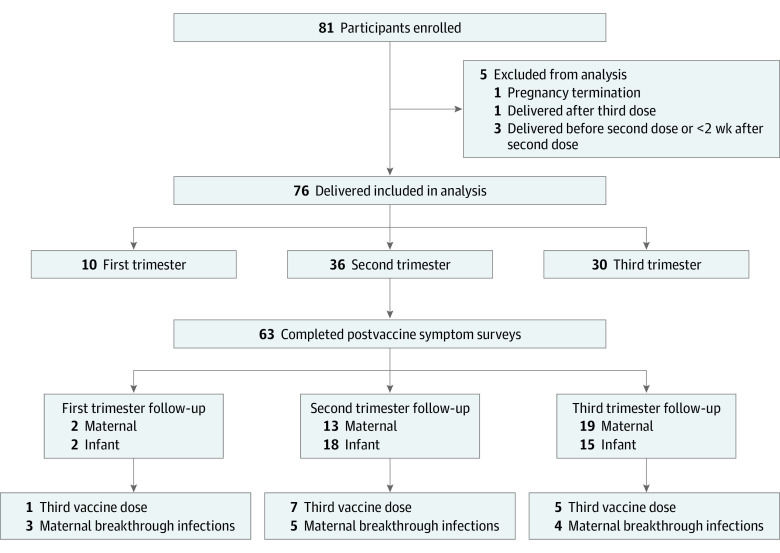
Participant Flowchart

All 81 participants who received their initial COVID-19 mRNA vaccination series during pregnancy were enrolled in this study between December 1, 2020, and December 31, 2021 (Figure 1). One participant was diagnosed as having a lethal fetal anomaly and underwent pregnancy termination at 20 weeks; this participant was subsequently excluded. We also excluded from final analyses 3 participants who delivered before second dose or less than 2 weeks after receiving the second vaccine dose, and 1 participant who delivered after the third vaccine dose. No participants had positive anti-SARS-CoV-2 antibodies prior to vaccination. The IgG titers are reported in eFigure 3 in Supplement 1.

Of 76 pregnant individuals (median [IQR] maternal age, 35 [29-41 years]) included in our complete analysis, 42 (55.3%) received the BNT162b2 vaccine and 34 (44.7%) received the mRNA-1237 vaccine; 28 (36.8%) were primigravid and 37 (48.7%) were nulliparous; 51 (67.1%) identified as White (Table). The median (IQR) gestational age at first vaccine dose was 22.8 (5.4-33.9) weeks. There were no differences in maternal characteristics between the 2 groups. Most participants received the third vaccine dose postpartum.

**Table.** Sample Characteristics Overall and by Vaccine Manufacturer

Maternal characteristic	Full cohort (n = 76)	BNT162b2 (n = 42 [55.3%])	mRNA-1237 (n = 34 [44.7%])	*P* value
Maternal age, median (IQR), y	35 (29-41)	34 (32-39)	35 (29-40)	.57
Primigravid, No. (%)	28 (36.8)	42 (33.3)	34 (41.2)	.48
Previous births				
Nulliparous, No. (%)	37 (48.7)	20 (47.6)	17 (50.0)	.84
Median (IQR) No. of births	1.0 (0.0-2.0)	1.0 (0.0-2.0)	0.5 (0.0-1.0)	.78
Race and ethnicity, No. (%)				
Asian	8 (10.5)	4 (9.5)	4 (11.8)	.43
Black or African American	2 (2.6)	1 (2.4)	1 (2.9)	
Hispanic or Latinx	4 (5.3)	4 (9.5)	0	
White	51 (67.1)	26 (61.9)	25 (73.5)	
>1 race	4 (4.1)	3 (7.1)	1 (2.9)	
Other/not specified^a^	7 (9.2)	4 (9.5)	3 (8.8)	
Prepregnancy BMI				
Patients with data, No.	59	33	26	
Median (IQR)	21.9 (18.6-30.2)	22.4 (19.6-29.0)	21.6 (19.5-25.1)	.26
Gestational age in weeks at first vaccine dose.				
Patients with data, No.	76	42	34	
Median (IQR), wk	22.8 (5.4-33.9)	23.6 (8.6-33.1)	21.3 (12.4-31.6)	.76
Postpartum weeks at third vaccine dose (by dose 3 type)^b^				
Patients with data, No.	45	26	19	
Median (IQR), wk	21.3 (3.4-34.9)	17.1 (3.4-26.6)	24.7 (15.1-32.9)	.007
Time after vaccination that symptoms were assessed				
Dose 1				
Patients with data, No.	61	33	28	
Median (IQR), d	141 (88-251)	138 (103-206)	174 (100-197)	.99
Dose 2				
Patients with data, No.	60	32	28	
Median (IQR), d	123.54 (60-362)	110.5 (82-185)	154.5 (75-223)	.72
Dose 3 (by dose 3 type)				
Patients with data, No.	25	15	10	
Median (IQR), d	105 (35-143)	114 (83-143)	73.54 (35-95)	.01

Abbreviation: BMI, body mass index (calculated as weight in kilograms divided by height in meters squared).

^a^ Other included not specified or did not state race and ethnicity.

^b^ Of 26 pregnant individuals who received the third BNT162b2 dose, 5 had received mRNA-1237 dose 1 and 2; of 19 pregnant individuals who received the third mRNA-1237 dose, 4 had received BNT162b2 dose 1 and 2.

### COVID-19 mRNA Vaccination–Related Symptoms

Participants were asked to report their postvaccination symptoms (eTable 1 in Supplement 1). Of 76 participants, the survey response rate was 78.9% (60 respondents) for vaccine dose 1, 76.3% (58 respondents) for vaccine dose 2, and 32.9% (25 respondents) for vaccine dose 3. Most participants reported local (eg, injection-site tenderness, erythema) or generalized (eg, fever, chills, headaches, nausea or vomiting, and muscle or body aches) postvaccination symptoms. Generalized symptoms were more common after the second dose than after the first dose (42 of 59 [71.2%] vs 26 of 59 [44.1%]; *P* = .007). Recipients of mRNA-1237 reported more generalized symptoms than recipients of BNT162b2 after the second (25 of 27 [92.6%] vs 17 of 32 [53.1%]; *P* = .001) and third (9 of 10 [90.0%] vs 7 of 15 46.7%; *P* = .04) doses. There were no serious adverse events after vaccination in the cohort.

### Longitudinal SARS-CoV-2 Vaccination Antibody Response During Pregnancy

We evaluated SARS-CoV-2 IgG antibody titers over time (eFigure 1 in Supplement 1). Vaccination in all trimesters elicited a robust antibody response that persisted through delivery. There was no difference in IgG titers among 10 individuals vaccinated in the first trimester and 36 individuals in the second trimester. The IgG titer among 30 individuals vaccinated in the third trimester (median [IQR], 2013 [1426-4062] RFU) was higher than that among individuals vaccinated in the first (median [IQR], 305 [199-425] RFU; *P* < .001) and second (median [IQR], 894 [443-1381] RFU; *P* = .02) trimesters (eFigure 1A in Supplement 1). Patterns in antibody titer decay were similar in all groups (eFigure 1B in Supplement 1). The 30-day rate of antibody decline was nearly identical for mRNA-1273 (mean [SD], −24.6% [7.3%] per month; n = 15) and BNT162b2 (−23.5% [7.1%] per month; n = 22; *P* = .65).

### SARS-CoV-2 Vaccine-Related Transplacental IgG Transfer

**Figure 2.**
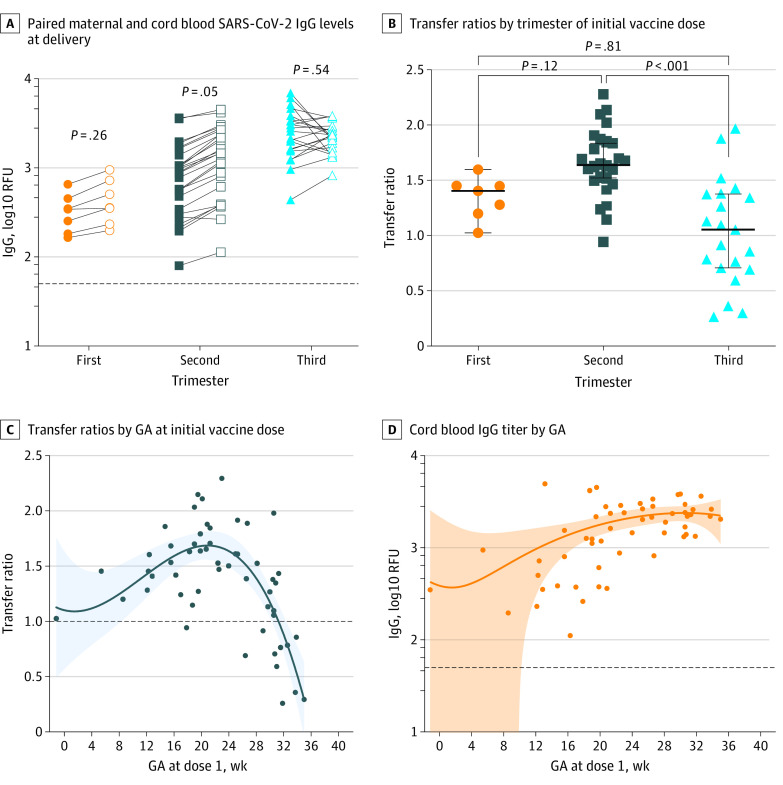
Transplacental Transfer of Vaccine-Induced SARS-CoV-2 immunoglobulin G (IgG) Antibodies at Delivery A, Solid shapes indicate maternal SARS-CoV-2 IgG antibody levels at delivery, and open shapes indicate cord blood SARS-CoV-2 IgG antibody levels. The horizontal dotted line represents positive cutoff value of 50 relative fluorescent units (RFU). B, Transfer ratios were defined as cord blood IgG titer divided by maternal IgG titer, by trimester of initial vaccine dose. Heavy and light horizontal lines represent median value and interquartile ranges, respectively. Values of *P* < .001 by Kruskal-Wallis test with Dunn multiple comparisons. C, Curved line represents the interpolation of values, with shaded areas representing interquartile range. The horizontal dotted line represents a transfer ratio of 1.0. D, Curved line represent the interpolation of values, with shaded areas representing interquartile range. The horizontal dotted line represents positive cutoff value of 50 RFU. GA indicates gestational age.

All trimester groups exhibited transplacental IgG transfer at the time of delivery (Figure 2). There was no difference between maternal and cord blood IgG titers at delivery in the first and third trimester groups. Individuals vaccinated in the second trimester had higher median cord blood IgG titers (1629 RFU) than maternal IgG titers (894 RFU; *P* = .05) (Figure 2A). Individuals who delivered less than 2 weeks after the second vaccine dose had low maternal and cord IgG titers at delivery (eFigure 1 and eFigure 2 in Supplement 1).

There was no difference in median transfer ratios between individuals vaccinated in the first and second trimesters and between individuals vaccinated in the first and third trimesters (Figure 2B). The median transfer ratio was higher among individuals vaccinated in the second trimester, and highest among individuals vaccinated between 20 and 24 weeks’ gestation (Figure 2C), although absolute cord blood IgG titers at the time of delivery were similar for all individuals vaccinated after 20 weeks’ gestation (Figure 2D). Notably, all infants’ cord blood had IgG titers above the positive cutoff value regardless of the trimester of vaccination.

### Reactogenicity and Immunogenicity of COVID-19 mRNA Vaccination in Pregnancy

**Figure 3.**
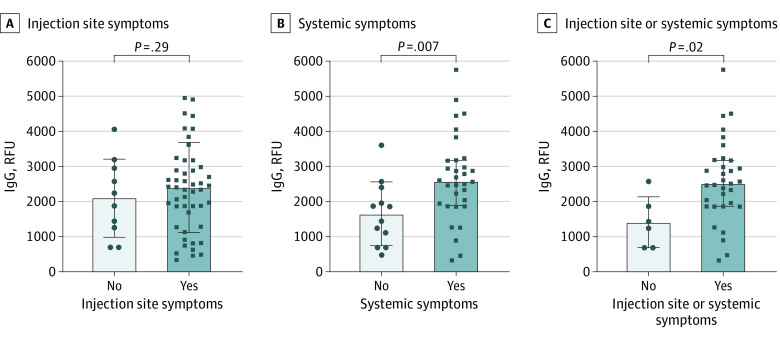
Symptoms and IgG Titers After Second Vaccine Dose and Immunoglobulin G Titer Measurement A, Injection site symptoms included soreness, redness, or itching localized to the injection site. B, Systemic symptoms included fatigue, fever or chills, body aches or joint pain, headache, and nausea or vomiting. Box limits indicate means; horizontal lines represent SEs.

We evaluated the association between postvaccination symptoms and maternal IgG titers. There was no difference in median IgG titers after the second dose among individuals with or without local injection-site symptoms. Individuals with systemic postvaccination symptoms had 65.6% higher median IgG titers and median peak IgG titers after the second dose than individuals who did not experience systemic postvaccination symptoms (eTable 2 in Supplement 1; Figure 3). In individuals with any site or systemic symptoms after dose 2, median cord IgG titers were 6.3-fold higher than in individuals without symptoms. The presence of local or systemic symptoms after dose 1 or 2 was not associated with gestational age at first vaccine dose or with the transplacental IgG transfer ratio.

### Perinatal Outcomes

There were no differences in most perinatal outcomes between individuals who received either mRNA vaccine (eTable 3 in Supplement 1) although there was a difference in rates of preterm deliveries. The overall preterm delivery rate in our cohort was 5.3% (4 of 76 deliveries); all preterm deliveries were among mRNA-1237 recipients. Of those deliveries, 2 (50.0%) were spontaneous and 2 were medically indicated (1 scheduled cesarean for vasa previa at 34 weeks and 1 induction of labor for preeclampsia with severe features at 32 weeks).

Five pregnancies were affected by fetal anomalies (6.6%). Details regarding specific anomalies and timing of vaccination are given in eTable 4 in Supplement 1.

### Longitudinal Infant Antibody Kinetics and Clinical Outcomes

**Figure 4.**
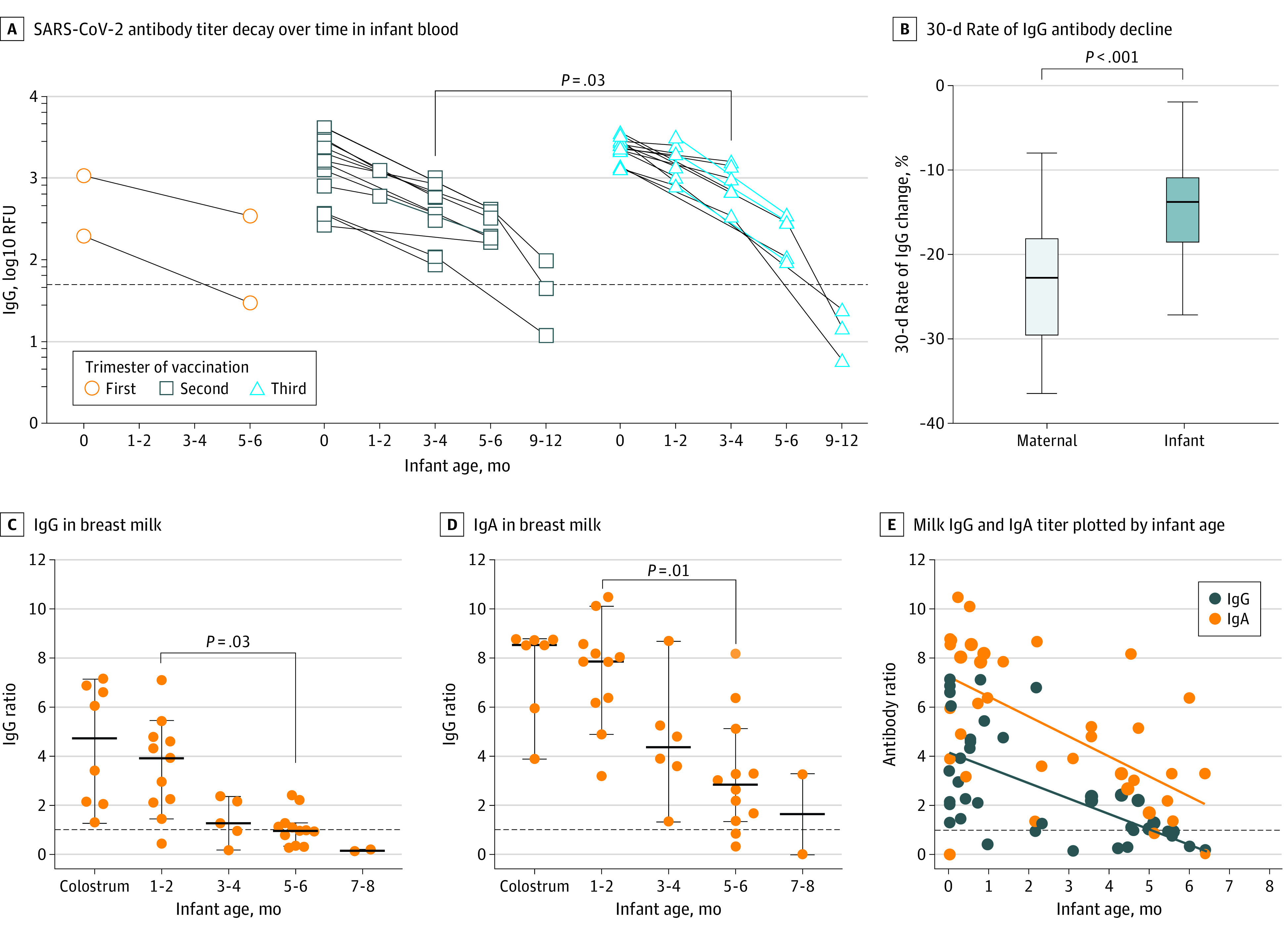
SARS-CoV-2 Antibody Titer Decay Over Time in Infant Blood and Milk of Vaccinated Mothers A, Infant blood was obtained at the indicated intervals after delivery up to 12 months of age; titer patterns are grouped by trimester of maternal vaccination. Median values at each time point were compared between second and third trimester groups using Mann-Whitney *U* tests. The horizontal dotted line represents positive cutoff value of 50 relative fluorescent units (RFU). B, Boxes represent IQRs; heavy horizontal lines, medians, and light horizontal lines the positive threshold for the assay. C and D, Milk from mothers who were fully vaccinated with 2 doses before delivery was collected after delivery and grouped by infant age in months. IgG (C) and IgA (D) in breast milk were detected by enzyme-linked immunosorbent assay. Median values were compared among different time points using Kruskal-Wallis test with Dunn multiple comparison. The heavy horizontal dotted lines represent positive cutoff value of ratio 1, and the light horizontal lines represent the positive threshold for the assay. E, Milk IgG and IgA titers plotted by infant age in months. Simple linear regression lines are shown for IgG and IgA titers. No significant difference was observed between the 2 slopes. Horizontal dotted line represents positive cutoff value of a ratio of 1.

We measured maternally derived infant antibody levels up to 12 months of age to investigate the kinetics of antibody durability in these infants. No infants had received COVID-19 vaccinations at the time their blood samples were obtained. Figure 4A shows SARS-CoV-2 antibody titer decay in infants born to vaccinated mothers, analyzed by trimester of maternal vaccination. Among most infants of mothers vaccinated in the second and third trimesters, IgG titers remained positive for at least 5 to 6 months. The 30-day rates of antibody decline for infants (mean [SD], −15.7% [6.0%] per month [n = 16] for mRNA-1273 and −12.9% [4.3%] per month [n = 12] for BNT162b2) were significantly slower than their mothers’ rates (mean [SD] mRNA-1273, −24.6% [7.3%] per month [n = 15]; mean [SD] BNT162b2, −23.5% [7.1%] per month [n = 22]; *P* < .001 for both) (Figure 4B).

Median IgG titers were higher at 3 to 4 months among infants of individuals vaccinated in the third trimester compared with the second trimester (median [IQR], 851 [611-1465] vs 356 [109-655] RFU; *P* = .03). Nine infants developed mild COVID-19 infections from December 1, 2021, through March 31, 2022, co-occurring with mild maternal infection in all but 1 case. The mean (range) infant age at COVID-19 infection was 6.9 (5.0-8.7) months (eTable 5 in Supplement 1). Of the 11 mothers who had COVID-19 illness postpartum, 8 infants (72.7%) also tested positive for COVID-19, including 1 set of twins. The incidence of infant COVID-19 infection was not associated with maternal site or systemic symptoms after vaccine dose 2; there was no difference in mean cord IgG titers between infants who did vs did not develop COVID-19 infection.

One second-trimester participant delivered monochorionic-diamniotic twins at 32 weeks due to preeclampsia with severe features. Maternal and cord blood titers in both twins were comparable to those observed in singletons in our cohort. Their IgG titers through 10 months of life are given in eFigure 3 in Supplement 1. The twins had nearly identical IgG titers until 7 months of life. At 9 months, both had mild COVID-19 infections. Their IgG titers were again determined approximately 1 month after infection; one twin’s IgG titer remained above the limit of detection, and the other twin’s titer was no longer positive despite their recent infection.

### Antibody Transfer in Human Milk

We investigated the persistence of milk-related antibodies in mothers vaccinated during pregnancy. Median IgG titers were significantly higher at 1 to 2 months than at 5 to 6 months (Figure 4C and 4D) and remained above the positive cutoff for at least 5 to 6 months after birth; IgA titers were higher than IgG titers in milk although there was no difference in the rate of decay between the them (Figure 4B; *P* = .40).

### Third Dose Boosters and Breakthrough COVID-19 Infections

In total, 45 participants (59.2%) received a booster (third dose) after delivery (median [IQR], 36.4 [27.8-40.4] weeks after receipt of dose 2). We obtained blood samples from 10 participants (13.2%). Twelve individuals (15.7%) experienced a breakthrough infection (median [IQR], 44.9 [38.4-48.1] weeks after receipt of dose 2). Additional blood samples were collected from those participants 4 to 8 weeks after receipt of dose 3 or breakthrough infection. In eFigure 4 in Supplement 1, we show the individual longitudinal IgG titer trajectories of each participant. In all trimesters of initial vaccination, a third vaccine dose or COVID-19 infection elicited increases in IgG titers. There was no difference in mean IgG titers after receipt of dose 3 vs after COVID-19 infection (3629 vs 4557 RFU; *P* = .34). The rate of maternal COVID-19 infection during the Omicron wave in early 2022 was 16%, all after vaccine dose 3.

## Discussion

This cohort study provides a detailed report on longitudinal antibody responses and symptoms after COVID-19 mRNA vaccination in all trimesters of pregnancy. We found that receipt of mRNA vaccines provoked robust immune responses in pregnant individuals. Rates of postvaccination symptoms were similar to those in the v-safe pregnancy registry^4^ and in our prior evaluation of mRNA vaccination in a lactation cohort,^21^ but higher than those described in clinical trials.^27,28^ There were no serious adverse events in either the mRNA-1273 or the BNT162b2 vaccine group, consistent with abundant data that COVID-19 vaccines are safe in pregnancy. The IgG transfer ratios were highest following second trimester vaccination. Maternally derived antibodies persisted until at least 5 to 6 months of life in most infants regardless of trimester of maternal vaccination. The rate of antibody decay per month among vaccinated pregnant individuals was comparable to that of nonpregnant adults reported elsewhere,^26^ but significantly more rapid than the rate of antibody decay of their offspring. Median IgG and IgA titers remained positive in milk samples up to 5 to 6 months postpartum although there was a significant decrease in both between the 1 to 2–month and 5 to 6–month tests.

Few studies have evaluated the association between COVID-19 mRNA vaccine reactogenicity and immunogenicity in pregnant individuals. Individuals who reported symptoms, particularly generalized symptoms such as fever, had higher mean IgG titers after the second dose and mean peak IgG titers, similar to findings from a separate cohort.^29^ We found that mothers with systemic symptoms had higher mean cord blood IgG levels than mothers without symptoms. This finding suggests that systemic postvaccination symptoms are associated with a more robust immune response, resulting in higher antibody titers in both mothers and infants. Reports for nonpregnant individuals have shown varying associations between reactogenicity and immunogenicity.^30,31,32,33,34^ Future research is needed to understand whether there are functional differences in these antibodies, and whether there are other differences in cell-mediated immune responses. In the meantime, our results may be useful when counseling pregnant individuals that individuals with or without postvaccination symptoms show an adequate response to the vaccine, and that systemic symptoms are not associated with adverse outcomes.

We reported follow-up to 12 months of age of infants born with vaccine-induced passive immunity to SARS-CoV-2, as we were able to in our cohort. This follow-up is of particular importance for counseling pregnant individuals about the benefits of vaccination, especially in the absence of an approved COVID-19 vaccine for infants less than 6 months of age. Maternal COVID-19 vaccination in pregnancy has been recently shown to be protective against COVID-19 infection-related hospitalizations in infants less than 6 months of age.^18^ The 30-day rates of antibody decline of −15.7% and −12.9% per month in our cohort for infants with mothers who received mRNA-1273 and BNT162b2, respectively, were higher than those in a previous study^35^ that reported a −4.3% 30-day decline from 78% to 52% at 6 months among infants of mothers with COVID-19 infections in pregnancy. Durability of vaccine- vs infection-induced antibodies has not been well studied in infants. The timing of infant COVID-19 infections in our cohort coincided with the typical age of waning maternally derived antibody levels, at approximately 6 months of age. Notably, all infant COVID-19 infections in our cohort occurred during December 1, 2021, through March 31, 2022, a time when the Omicron variant emerged and caused a large rise in infections in our area, around San Francisco, California, as in other parts of the United States. Vaccine efficacy during this period was low.^36^

The incidence of fetal anomalies in our cohort (6.6%) was higher than that of the general population (3.0%),^37^ due to referrals to our fetal treatment center for prenatally diagnosed anomalies. All participants with fetal anomalies were vaccinated after completion of embryogenesis (ie, in the second or third trimester), supporting that the presence of anomalies was independent of maternal vaccination. Other population-based pregnancy cohorts have reported no increased incidence of birth defects after maternal vaccination in any trimester.^4,38,39,40,41^

### Strengths and Limitations

A strength of our study was the lack of COVID-19 infection in pregnancy (or in the prenatal period) in our cohort, allowing for delineation of specific vaccine-related effects, rather than effects of prior infection. As COVID-19 infection rates continue to increase, this lack of infection among participants will prove difficult in future studies. In addition, we provided longitudinal outcomes of the maternal-infant dyad up to 12 months of the infant’s life. All but 1 infant COVID-19 infection coincided with maternal infections.

Limitations of this study included low response rate to symptom surveys and long response times for individuals who did respond, introducing recall and sample biases. More timely responses would have clarified the association between postvaccination symptoms and the immune response. Our interpretation of immune response focused on IgG titers in blood and milk and IgA titers in milk rather than assessment of a more comprehensive immune profile inclusive of antibody function and cell-mediated responses. Other limitations included a lack of racial and ethnic diversity in our cohort and restriction to a single metropolitan area although these factors were unlikely to influence biological responses to vaccination.

## Conclusions

The findings of this cohort study indicated that vaccination with mRNA COVID-19 vaccines in pregnancy provoked a robust immune response for the mother-infant dyad for approximately 6 months after birth. Systemic symptoms after receipt of a vaccine dose may indicate a more robust immune response, as measured by higher antibody titers. The association between postvaccination symptoms and magnitude of immune response warrants further study.

## Notes

Supplement 1.
eMethods.
**eTable 1.** Symptoms After Each Vaccine Dose **eTable 2.** IgG Antibody Levels by Symptom Experience After Second Vaccine Dose **eTable 3.** Perinatal Outcomes by Vaccine Manufacturer **eTable 4.** Fetal Anomalies and Vaccination Details **eTable 5.** Infant COVID-19 Infection **eFigure 1.** Maternal SARS-CoV-2 IgG Levels After Vaccination During Pregnancy **eFigure 2.** Paired Maternal SARS-Cov-2 IgG Antibody Levels at Delivery and Cord Blood SARS-Cov-2 IgG Antibody Levels **eFigure 3.** Longitudinal SARS-CoV-2 IgG Titer in a Pair of Twins After Birth **eFigure 4.** Longitudinal Maternal Antibody Titers in Participants Who Received Third Dose and/or COVID-19 Infection

Supplement 2.
Data Sharing Statement
