# Supplementary material for: Assessment of Adverse Reactions, Antibody Patterns, and 12-month Outcomes in the Mother-Infant Dyad After COVID-19 mRNA Vaccination in Pregnancy
Source: JAMA Netw Open. 2023 Jul 14;6(7):e2323405. doi: 10.1001/jamanetworkopen.2023.23405 (PMC10349345; doi:10.1001/jamanetworkopen.2023.23405)
Supplement: Supplement 1. — eMethods. eTable 1. Symptoms After Each Vaccine Dose eTable 2. IgG Antibody Levels by Symptom Experience After Second Vaccine Dose eTable 3. Perinatal Outcomes by Vaccine Manufacturer eTable 4. Fetal Anomalies and Vaccination Details eTable 5. Infant COVID-19 Infection eFigure 1. Maternal SARS-CoV-2 IgG Levels After Vaccination During Pregnancy eFigure 2. Paired Maternal SARS-Cov-2 IgG Antibody Levels at Delivery and Cord Blood SARS-Cov-2 IgG Antibody Levels eFigure 3. Longitudinal SARS-CoV-2 IgG Titer in a Pair of Twins After Birth eFigure 4. Longitudinal Maternal Antibody Titers in Participants Who Received Third Dose and/or COVID-19 Infection [file jamanetwopen-e2323405-s001.pdf]

Supplementary Online Content

Cassidy AG, Li L, Golan Y, et al. Assessment of adverse reactions, antibody patterns, and 12-month outcomes in the mother-infant dyad after COVID-19 mRNA vaccination in pregnancy. *JAMA Netw Open*. 2023;6(7):e2323405. doi:10.1001/jamanetworkopen.2023.23405

eMethods.
eTable 1. Symptoms After Each Vaccine Dose
eTable 2. IgG Antibody Levels by Symptom Experience After Second Vaccine Dose
eTable 3. Perinatal Outcomes by Vaccine Manufacturer
eTable 4. Fetal Anomalies and Vaccination Details
eTable 5. Infant COVID-19 Infection
eFigure 1. Maternal SARS-CoV-2 IgG Levels After Vaccination During Pregnancy
eFigure 2. Paired Maternal SARS-Cov-2 IgG Antibody Levels at Delivery and Cord Blood SARS-Cov-2 IgG Antibody Levels
eFigure 3. Longitudinal SARS-CoV-2 IgG Titer in a Pair of Twins After Birth
eFigure 4. Longitudinal Maternal Antibody Titers in Participants Who Received Third Dose and/or COVID-19 Infection
eReferences.

This supplementary material has been provided by the authors to give readers additional information about their work.

© 2023 Cassidy AG et al. *JAMA Network Open*.

eMethods.

Definition of clinical variables

Hypertensive disorders of pregnancy (pre-eclampsia and/ gestational hypertension) were diagnosed by the prenatal care provider based on the American College of Obstetricians and Gynecologists' (ACOG) criteria. Gestational hypertension was defined as a systolic blood pressure of 140 mm Hg and/or a diastolic blood pressure of 90 mm Hg on two occasions at least 4 hours apart after 20 weeks' gestation in a woman parturient with a previously normal blood pressure. Pre-eclampsia was defined as the above blood pressure criteria plus proteinuria (at least 300 mg in a 24-hour urine collection, or protein-to-creatinine ratio of 0.3), or, in the absence of proteinuria, new thrombocytopenia (platelet count less than 100 x 10^9/L), renal insufficiency (serum creatinine greater than 1.1 mg/dL or a doubling of the baseline serum creatinine in the absence of other renal disease), impaired liver function (elevated serum liver transaminases to twice normal concentration), pulmonary edema, or new-onset headache or vision changes unresponsive to treatment and unexplained by other diagnoses. Gestational diabetes mellitus (GDM) is diagnosed using a two-step approach at our institution: patients first receive a 50-gram oral glucose solution followed by a 1-hour venous glucose level; if this exceeds our screening threshold, the patient receives a 100-gram, 3-hour oral glucose tolerance test (OGTT). GDM is diagnosed by two or more abnormal values on the 3-hour OGTT. Chorioamnionitis, or suspected intrapartum intraamniotic infection, was diagnosed by the intrapartum care team based on ACOG criteria: maternal temperature ≥ 39.0 degrees Celsius, or maternal temperature 38.0-38.9 degrees Celsius and one additional clinical risk factor (maternal leukocytosis, purulent cervical drainage, or fetal tachycardia).

Post-vaccine surveys

After your FIRST/SECOND/THIRD dose of the vaccine, did you have a fever or feel feverish? Yes/No

When after your FIRST/SECOND/THIRD dose of the COVID-19 vaccine did you start feeling feverish?
0-24 hours after my first/second/third vaccine shot
24-48 hours after my first/second/third vaccine shot
48-72 hours after my first/second/third vaccine shot
More than 72 hours, but less than 1 week after my first/second/third vaccine shot
More than 1 week after my first/second/third vaccine shot

What was the highest temperature that you measured after your FIRST/SECOND/THIRD vaccine dose?

After your FIRST/SECOND/THIRD vaccine dose, did you have any of the following symptoms where you got the shot (injection site)?
Pain
Redness
Swelling
Itching
None

When did these symptoms where you got your FIRST/SECOND/THIRD vaccine shot start?
0-24 hours after my first/second/third vaccine shot
24-48 hours after my first/second/third vaccine shot
48-72 hours after my first/second/third vaccine shot
More than 72 hours, but less than 1 week after my first/second/third vaccine shot
More than 1 week after my first/second/third vaccine shot

Did you experience any of these symptoms after your FIRST/SECOND/THIRD dose of COVID-19 vaccine?
Chills
Headache
Joint pains
Muscle aches or body aches
Fatigue or tiredness
Nausea
Vomiting

© 2023 Cassidy AG et al. *JAMA Network Open*.

Diarrhea
Abdominal pain
Rash in the immediate area surrounding the injection point
Rash not in the immediate area surrounding the injection point
Lump or swelling in breast on the same side as vaccine administration
Lump or swelling on the opposite side of vaccine administration
Mastitis
Decreased milk supply
Any other symptoms or health conditions
None

Please specify the other symptoms you have experienced after your FIRST/SECOND/THIRD dose of the COVID-19 vaccine:

When after your FIRST/SECOND/THIRD vaccine dose did the symptom(s) you indicated above first start?
0-24 hours after my first/second/third vaccine shot
24-48 hours after my first/second/third vaccine shot
48-72 hours after my first/second/third vaccine shot
More than 72 hours, but less than 1 week after my first/second/third vaccine shot
More than 1 week after my first/second/third vaccine shot

Did any of the symptoms or health conditions you had after your FIRST/SECOND/THIRD vaccine dose cause you to:
Be unable to work
Be unable to do your normal activities
Get care from a doctor or other health care professional
None of the above

When after your FIRST/SECOND/THIRD dose of the vaccine did these experiences start?
0-24 hours after my first/second/third vaccine shot
24-48 hours after my first/second/third vaccine shot
48-72 hours after my first/second/third vaccine shot
More than 72 hours, but less than 1 week after my first/second/third vaccine shot
More than 1 week after my first/second/third vaccine shot

For how long did these experiences last?
Less than 24 hours
24-48 hours
48-72 hours
More than 72 hours, but less than 1 week 1 to 2 weeks
More than 2 weeks

Measurement of SARS-CoV-2 Specific IgM and IgG in Plasma Samples
Quartz glass probes pre-coated with either affinity-purified goat anti-human IgM (IgM capture) or Protein G (IgG capture) were dipped into diluted plasma samples, washed, then dipped into the assay reagent containing biotinylated, recombinant spike protein receptor binding domain (RBD) and nucleocapsid protein (NP). After washing, probes were incubated with Cy®5-streptavidin (Cy5-SA) polysaccharide conjugate reagent for cyclic amplification of the fluorescence signal. The background-corrected signal of SARS-CoV-2 specific IgM and IgG antibodies was reported as relative fluorescent units (RFU); measurements above 50 RFU were considered positive.

Measurement of SARS-CoV-2 Specific IgG and IgA in Milk Samples
Milk samples were either processed immediately by study staff or frozen by mothers in their home freezer immediately after pumping and transferred on ice to the lab for processing. Milk was aliquoted and stored at -80°C until analyzed. Anti-Spike ELISA assay (Euroimmune, Germany) was used to measure IgA and/or IgG levels in skim milk. Milk fat was separated by cold centrifugation, diluted 1:4 with the provided diluent buffer, and examined using the manufacturer’s protocol,[1,2] with an additional blocking step with 5% BSA in TBS with 0.5% Tween 20 for

© 2023 Cassidy AG et al. *JAMA Network Open*.

30 min before loading the samples as recommended to increase specificity. OD values of samples were calculated by dividing by the provided calibrator OD value; values with sample:calibrator ratio > 1 were considered positive. Milk samples were analyzed in duplicate.

© 2023 Cassidy AG et al. *JAMA Network Open*.

Supplemental tables:

eTable 1. Symptoms After Each Vaccine Dose

Symptoms	Full cohort				After 1st dose			After 2nd dose			After 3rd dose		
	1st dose	2nd dose	3rd dose	p[a]	BNT-162b2	mRNA-1237	p[b]	BNT-162b2	mRNA-1237	p[b]	BNT-162b2	mRNA-1237	p[b]
**Injection site symptoms, n (%)**	N=60	N=58	N=25		n=32	n=28		n=31	n=27		n=15	n=10	
Any	50 (83)	45 (78)	18 (72)	1v2: .22<br>1v3: .50<br>2v3: .99	27 (84)	23 (82)	.99	22 (71)	23 (85)	.23	10 (67)	8 (80)	.66
**Generalized symptoms, n (%)**	N=59	N=59	N=25		n=31	n=28		n=32	n=27		n=15	n=10	
Any	26 (44)	42 (71)	16 (64)	1v2: .007<br>1v3: .11<br>2v3: .99	11 (35)	15 (54)	.20	17 (53)	25 (93)	.001	7 (47)	9 (90)	.04
Fever/chills	2 (3)	20 (34)	8 (32)	1v2: <.001<br>1v3: .04<br>2v3: .99	0	2 (7)	.21	5 (16)	15 (56)	.002	3 (20)	5 (50)	.19
Muscle/body aches/joint pain	7 (12)	25 (42)	9 (36)	1v2: <.001<br>1v3: .008<br>2v3: .51	2 (6)	5 (18)	.24	8 (25)	17 (63)	.004	3 (20)	6 (60)	.09
Fatigue/headache	24 (41)	40 (68)	12 (48)	1v2: .007<br>1v3: .73<br>2v3: .22	11 (35)	13 (46)	.44	17 (53)	23 (85)	.01	5 (33)	7 (70)	.11
Nausea/vomiting/ diarrhea	0	8 (14)	2 (8)	1v2: .02<br>1v3: .50<br>2v3: .99	0	0	n/a	4 (13)	4 (15)	.99	0	2 (20)	.15

[a]The p-values in this column reflect McNemar's exact test.
[b]The p-values in this column reflect Fisher's exact

© 2023 Cassidy AG et al. *JAMA Network Open*.

eTable 2. IgG Antibody Levels by Symptom Experience After Second Vaccine Dose

**Symptom experience after dose 2**	N	IgG after dose 2 Median (IQR)	IgG in cord blood Median (IQR)
Any injection site symptoms?	45	p=0.29	p=0.32
Yes	34	2498 (1114, 4518)	2150 (364, 3789)
No	11	2233 (142, 2571)	1456 (1401, 2052)
Any systemic symptoms?	46	p=0.007	p=0.20
Yes	32	2596 (1840, 4455)	2220 (364, 3789)
No	14	1568 (1114, 2396)	1456 (879, 2203)
Any site or systemic symptoms?	45	p=0.02	p=0.03
Yes	39	2515 (1114, 4518)	2203 (364, 3789)
No	6	1335 (too few)	351 (too few)

© 2023 Cassidy AG et al. *JAMA Network Open*.

eTable 3. Perinatal Outcomes by Vaccine Manufacturer

Sample Characteristics	Full Cohort (N=76)	BNT162b2 (n=42)	mRNA-1237 (n=34)	p[a]
Gestational age at delivery				
Median (IQR)	39.3 (36.3, 41.0)	39.4 (37.7, 41.0)	39.2 (36.3, 40.4)	.060
Birth weight in kg				
Median (IQR)	3.32 (2.35, 4.25)	3.37 (2.88, 4.00)	3.24 (2.35, 4.06)	.17
Mode of delivery, n (%)				.55
Vaginal	63 (82.9)	36 (85.7)	27 (79.4)	
Cesarean	13 (17.1)	6 (14.3)	7 (20.6)	
Anomalies, n (%)	4 (5.3%)	1 (2.4%)	3 (8.8%)	.32
Apgar scores				
1 min, Mean (min, max)	7.6 (1, 9)	7.8 (1, 9)	7.5 (1, 9)	.52
5 min, Mean (min, max)	8.6 (4, 9)	8.6 (4, 9)	8.6 (5, 9)	.72
NICU admission, n (%)	11 (14.5)	5 (11.9)	6 (17.7)	.53
Pre-eclampsia/gHTN, n (%)	19 (25.0)	12 (28.6)	7 (20.6)	.60
GDM, n (%)	2 (2.6)	1 (2.4)	1 (2.9)	.99
Chorioamnionitis, n (%)	2 (2.6)	2 (4.8)	0	.50
SGA, n (%)	2 (2.6)	1 (2.4)	1 (2.9)	.99
Preterm birth, n (%)	4 (5.3)	0	4 (11.8)	.04
Induction of labor, n (%)	43 (56.6)	25 (59.5)	18 (52.9)	.64
Stillbirth, n (%)	0	0	0	n/a

© 2023 Cassidy AG et al. *JAMA Network Open*.

eTable 4. Fetal Anomalies and Vaccination Details

	**Vaccine manufacturer**	**GA at first vaccine dose (weeks)**	**Anomaly**	**Confirmed genetic diagnosis**
1	BNT162b2	13	Skeletal dysplasia	Yes
2	BNT162b2	30	Unilateral renal pelviectasis	Yes
3	mRNA-1237	20	Hypospadias, microphallus, bifid scrotum	Yes
4	mRNA-1237	35	Small muscular ventricular septal defect	No
5	mRNA-1237	29	Cleft lip	No

© 2023 Cassidy AG et al. *JAMA Network Open*.

eTable 5. Infant COVID-19 Infection

Infant age at COVID-19 diagnosis	N=9
Mean, SD (Months)	6.9, 1.48
Infant COVID-19 symptoms (n/%)	
Fatigue	5 (55.5)
Fever	7 (77.8)
Eye Redness	2 (22.2)
Swollen lymph nodes	1 (11.1)
Rash	1 (11.1)
Cough	7 (77.8)
Runny nose	8 (88.9)
Poor appetite	3 (33.3)
Insomnia	1 (11.1)
Diarrhea	2 (22.2)
Mild fussiness	1 (11.1)
Inspiratory stridor	1 (11.1)
How long did infant COVID-19 symptoms last (n/%)	
0-24 hours	2 (22.2)
48-72 hours	1 (11.1)
More than 72 hours, but less than one week	5 (55.5)
1-2 weeks	1 (11.1)
What type of medical support did your baby receive? (n/%)	
Did not need medical support or treatment	3 (33.3)
Consulted with infant's Pediatrician	6 (66.6)
Treated with medication	2 (22.2)
Medications administered: Dexamethasone, Tylenol	
Emergency Department visit	1 (11.1)
Hospitalization	0 (0)
Diagnosed with MIS-C (Multisystem Inflammatory Syndrome in Children)	0 (0)

© 2023 Cassidy AG et al. *JAMA Network Open*.

Supplemental figures:

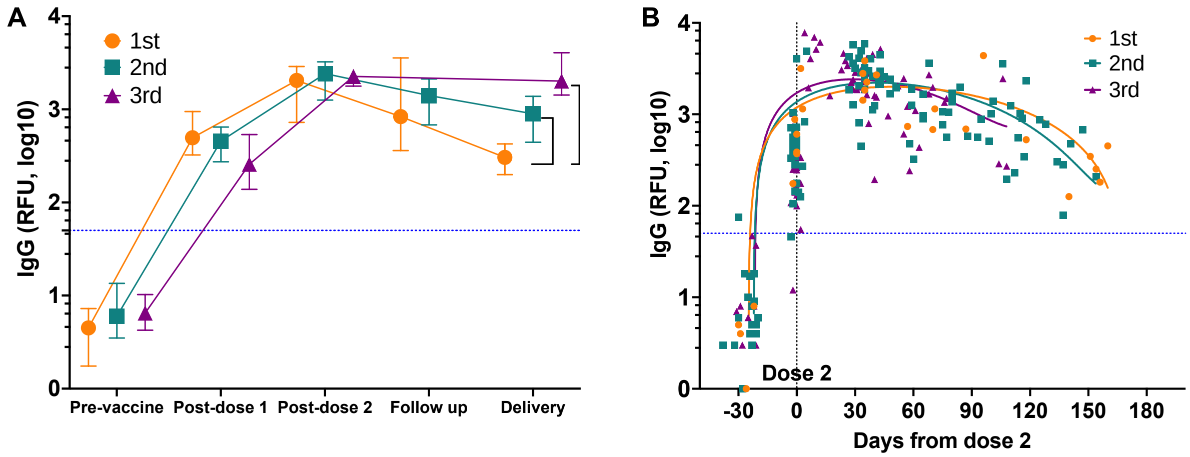

eFigure 1. Maternal SARS-CoV-2 IgG Levels After Vaccination During Pregnancy

- **A.** Comparisons of the median SARS-CoV-2 IgG antibody levels at each time point by trimester. Bars represent interquartile range for each time point. There was no difference between median IgG level at delivery for those vaccinated in the 1st and 2nd trimester. Horizontal dotted line represents positive cutoff value of 50 RFU.
- **B.** Individual SARS-CoV-2 IgG antibody values in relation to days since vaccine dose 2. Lines represent interpolation of trends by trimester. Horizontal dotted line represents positive cutoff value of 50 RFU.

© 2023 Cassidy AG et al. *JAMA Network Open*.

eFigure 2. Paired Maternal SARS-Cov-2 IgG Antibody Levels at Delivery and Cord Blood SARS-Cov-2 IgG Antibody Levels. "3 doses" group represents two participants who received their initial 2-dose mRNA vaccine series in the 1st trimester and received a 3rd dose prior to delivery. "Incomplete" represents two participants who had incomplete 2-dose vaccination series at the time of delivery.

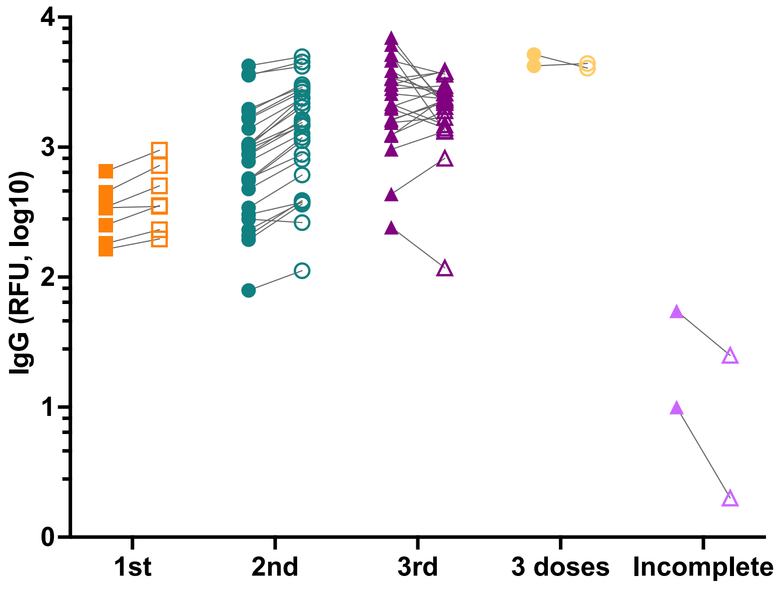

© 2023 Cassidy AG et al. *JAMA Network Open*.

eFigure 3. Longitudinal SARS-CoV-2 IgG Titer in a Pair of Twins After Birth

Monochorionic-diamniotic twins born 2.8 months after maternal vaccine dose 2. Vertical line marks the date of COVID-19 infection for both twins.

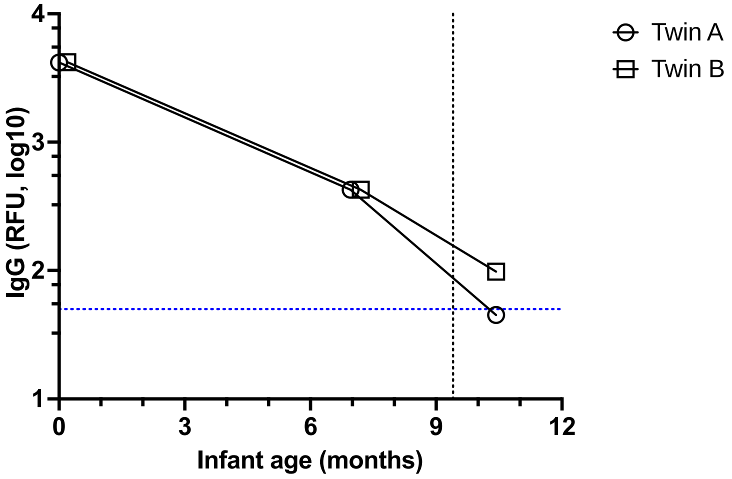

© 2023 Cassidy AG et al. *JAMA Network Open*.

eFigure 4. Longitudinal Maternal Antibody Titers in Participants Who Received Third Dose and/or COVID-19 Infection

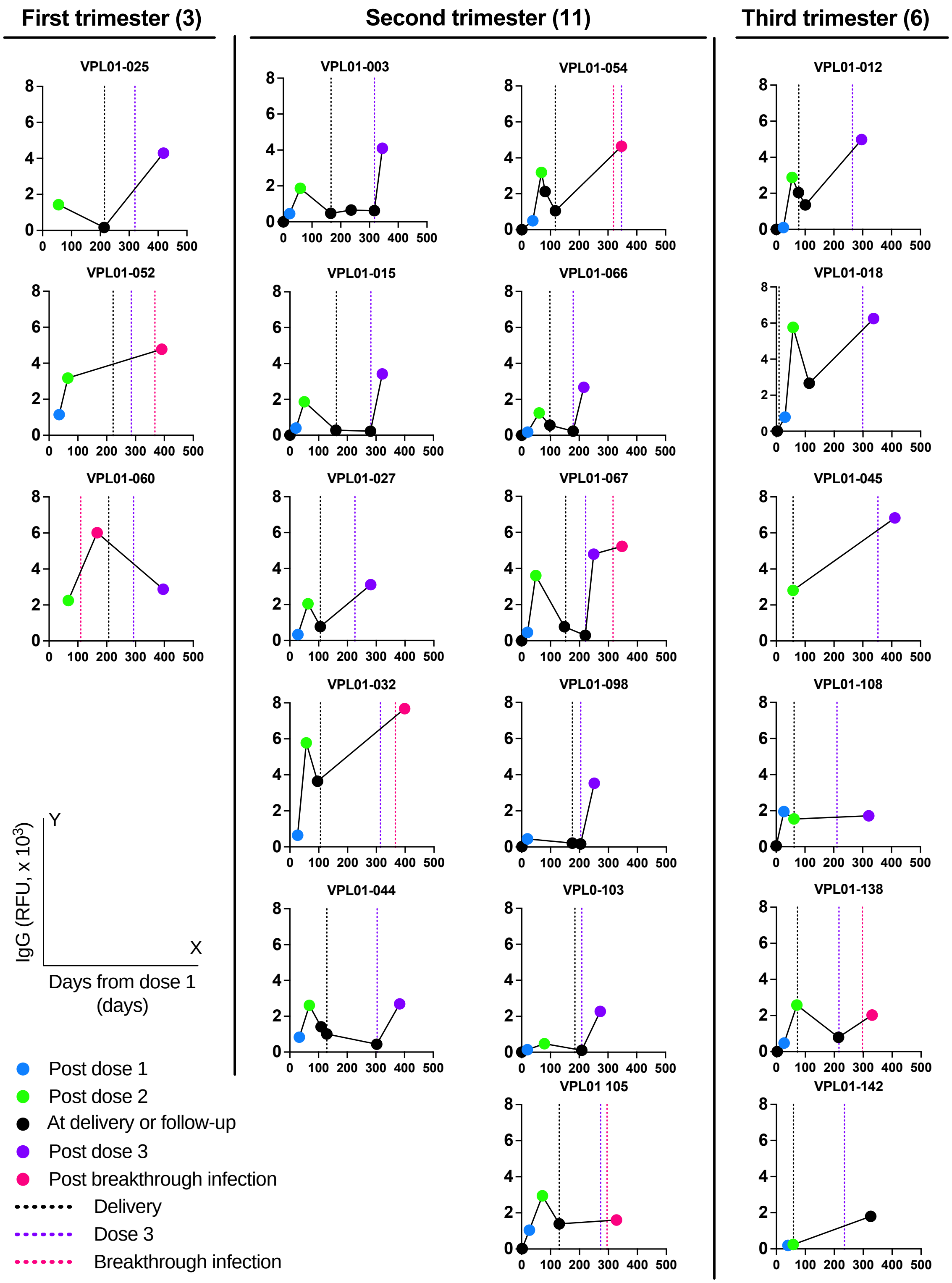

© 2023 Cassidy AG et al. *JAMA Network Open*.

eReferences

1. Golan Y, Prahl M, Cassidy AG, et al. COVID-19 mRNA Vaccination in Lactation: Assessment of Adverse Events and Vaccine Related Antibodies in Mother-Infant Dyads. *Frontiers in Immunology*. 2021;12. Accessed November 7, 2022. https://www.frontiersin.org/articles/10.3389/fimmu.2021.777103

2. Golan Y, Prahl M, Cassidy A, et al. Evaluation of Messenger RNA From COVID-19 BTN162b2 and mRNA-1273 Vaccines in Human Milk. *JAMA Pediatrics*. 2021;175(10):1069-1071. doi:10.1001/jamapediatrics.2021.1929

© 2023 Cassidy AG et al. *JAMA Network Open*.
