# Supplementary material for: Assessment of Adverse Reactions, Antibody Patterns, and 12-month Outcomes in the Mother-Infant Dyad After COVID-19 mRNA Vaccination in Pregnancy
Source: JAMA Netw Open. 2023 Jul 14;6(7):e2323405. doi: 10.1001/jamanetworkopen.2023.23405 (PMC10349345; doi:10.1001/jamanetworkopen.2023.23405)
Supplement: Supplement 2. — Data Sharing Statement [file jamanetwopen-e2323405-s002.pdf]

Data Sharing Statement

Cassidy. Assessment of Adverse Reactions, Antibody Patterns, and 12-month Outcomes in the Mother-Infant Dyad After COVID-19 mRNA Vaccination in Pregnancy. *JAMA Netw Open*. Published July 14, 2023. doi:10.1001/jamanetworkopen.2023.23405

Data
Data available: Yes
Data types: Deidentified participant data
How to access data: arianna.cassidy@ucsf.edu
When available: With publication

Supporting Documents
Document types: None

Additional Information
Who can access the data: Researchers whose proposed use of the data has been approved
Types of analyses: For a specified purpose
Mechanisms of data availability: After approval of a proposal and with a signed data access agreement
